# 4-Hydroxyestrone, an Endogenous Estrogen Metabolite, Can Strongly Protect Neuronal Cells Against Oxidative Damage

**DOI:** 10.1038/s41598-020-62984-y

**Published:** 2020-04-29

**Authors:** Hye Joung Choi, Anthony J. Lee, Ki Sung Kang, Ji Hoon Song, Bao Ting Zhu

**Affiliations:** 1 School of Life and Health Sciences, The Chinese University of Hong Kong, Shenzhen, Guangdong 518172 China; 2 Department of Pharmacology, Toxicology and Therapeutics, School of Medicine, University of Kansas Medical Center, Kansas City, KS 66160 USA

**Keywords:** Cell biology, Mechanisms of disease, Cellular neuroscience, Molecular medicine

## Abstract

Earlier studies showed that endogenous estrogens have neuroprotective effect against oxidative damage. The present study seeks to investigate the protective effect of various endogenous estrogen metabolites against oxidative neurotoxicity *in vitro* and *in vivo*. Using immortalized mouse hippocampal neuronal cells as an *in vitro* model, 4-hydroxyestrone, an estrone metabolite with little estrogenic activity, is found to have the strongest neuroprotective effect against oxidative neurotoxicity among 25 endogenous estrogen metabolites tested, and its protective effect is stronger than 17β-estradiol. Similarly, 4-Hydroxyestrone also exerts a stronger protective effect than 17β-estradiol against kanic acid-induced hippocampal oxidative damage in rats. Neuroprotection by 4-hydroxyestrone involves increased cytoplasmic translocation of p53 resulting from SIRT1-mediated deacetylation of p53. Analysis of brain microsomal enzymes shows that estrogen 4-hydroxylation is the main metabolic pathway in the central nervous system. Together, these results show that 4-hydroxyestrone is an endogenous neuroestrogen that can strongly protect against oxidative neuronal damage.

## Introduction

Human epidemiological studies have suggested that early start of estrogen replacement therapy in postmenopausal women is associated with a reduction in neurodegenerative diseases and improvements in cognition and memory^1–4^. In support of these human observations, many *in vitro* studies have shown that estrogens have a protective effect in neuronal cells against certain types of insults^5–17^. In addition, it was reported that estrogen therapy is clinically associated with an improved recovery from ischemic stroke^18,19^. The protective effect of estrogens in ischemic stroke has also been demonstrated in several acute cerebral ischemia animal models involving rats, mice and gerbils^20–25^.

Oxidative stress is recognized as an important etiological factor in development of neurodegenerative diseases (reviewed in ref. ^26–28^). Neuronal oxidative stress can be caused by a number of cellular mechanisms^29^. Glutamate, an excitatory neurotransmitter, is neurotoxic when present at high concentrations, in part via the induction of cellular oxidative stress. Glutamate-induced oxidative toxicity has been described in neuronal cell lines^30^, primary neuronal cultures^31^, and oligodendrocytes^32^. Glutamate-induced oxidative stress and neuronal death are considered an important contributing factor in neurodegenerative diseases, which occurs before the onset of significant pathological change and clinical symptoms^33^.

Mechanistically, the glutamate-induced oxidative cytotoxicity is mediated via two different pathways, namely, the receptor-mediated excitotoxicity and the non-receptor-mediated oxidative cytotoxicity^33–36^. In the former case, the glutamate-induced excitotoxicity results from activation of ionotropic glutamate receptors, which subsequently leads to transient Ca^2+^ fluxes, increased levels of reactive oxygen species (ROS), and ultimately cell death. In the latter case, the glutamate-induced oxidative stress is caused by an inhibition of the glutamate-cystine antiporter and subsequent promotion of cystine efflux and/or blockade of cystine uptake, which then results in reduction of intracellular glutathione and accumulation of intracellular ROS, and ultimately, oxidative cytotoxicity^35–38^.

In the present study, we seek to investigate the protective effect of various endogenous metabolites of 17β-estradiol (E_2_) and estrone (E_1_) against oxidative neurotoxicity using both *in vitro* and *in vivo* models, and also to determine the mechanism underlying their neuroprotective action. We find that 4-OH-E_1_, an endogenous E_1_ metabolite with little estrogenic activity, has the strongest protective effect against glutamate-induced oxidative neurotoxicity *in vitro* and kainic acid-induced neuronal damage *in vivo*. Analysis of brain microsomal enzymes showed that estrogen 4-hydroxylation is the main metabolic pathway in the central nervous system. Mechanistically, the neuroprotection by 4-OH-E_1_ involves increased cytoplasmic localization of p53 resulting from SIRT1-mediated p53 deacetylation.

## Results

### 4-Hydroxyestrogens have strongest neuroprotective effect *in vitro* and *in vivo*

**Figure 1:**
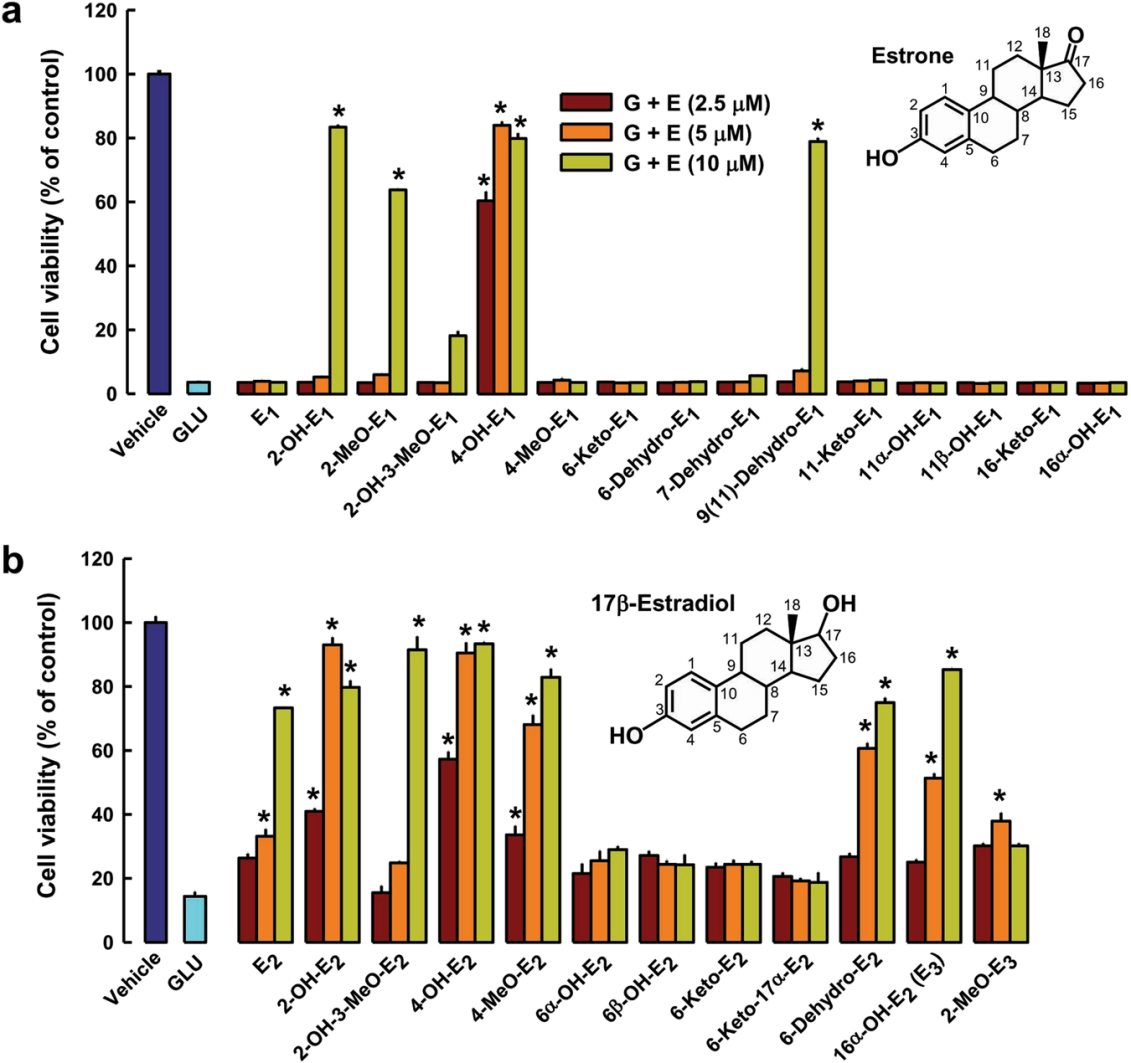
Protective effect of E_1_, E_2_, and many of their endogenous metabolites against glutamate (GLU or G)-induced oxidative cell death in HT22 hippocampal neuronal cells. **(a)** The neuroprotective effect of E_1_ and its metabolites (the inset is the structure of E_1_). **(b)** The neuroprotective effect of E_2_ and its metabolites (the inset is the structure of E_2_). Cells are exposed to 5 mM glutamate for 24 h, and the estrogen (at three selected concentrations as shown) is introduced at the same time as glutamate. Note that cells in the control group are treated with vehicle only, whereas all other groups (including the estrogen groups) are treated with 5 mM glutamate. Cell viability is estimated using the MTT assay. Each value is mean ± S.D. of 6‒8 replicate measurements. *P < 0.05 versus the glutamate alone group.

Using HT22 hippocampal neurons as an *in vitro* model, we compare the protective effect of E_1_, E_2_, and 25 of their endogenous metabolites against glutamate-induced oxidative neurotoxicity. The initial screening using the MTT assay shows that the presence of some of these estrogen metabolites exerts varying degrees of protection against glutamate-induced oxidative neurotoxicity (Fig. 1). Clearly, 4-OH-E_1_ and 4-OH-E_2_ have strongest neuroprotective effect, which is markedly stronger than their respective parent hormones, E_1_ and E_2_. The strong neuroprotective effect of 4-OH-E_1_ is of particular interest, as this endogenous estrogen metabolite is almost completely devoid of binding affinity for the ERα and ERβ^39^. Some additional data for the *in vitro* neuroprotective effect of this estrogen metabolite against glutamate-induced cytotoxicity are shown in Supplementary Fig. 1. The neuroprotective effect of 4-OH-E_1_ is also confirmed using other cell death parameters, including gross morphological changes (Supplementary Fig. 2a, upper two panels), TUNEL-positive cells (Supplementary Fig. 2a, lower panel; Supplementary Fig. 2b), and DNA fragmentation (Supplementary Fig. 2c). In addition, flow cytometric analyses of HT22 neuronal cells stained with PI alone or double-stained with annexin-V and PI show that co-treatment with 4-OH-E_1_ almost completely abrogates glutamate-induced cell death while the cell cycle is not altered (Supplementary Fig. 3a,b). It is of note that 4-OH-E_1_ (at 3 and 5 μM) does not appear to have a significant restorative effect in cultured HT22 cells that are pretreated with glutamate for 12 or 24 hours.

**Figure 2:**
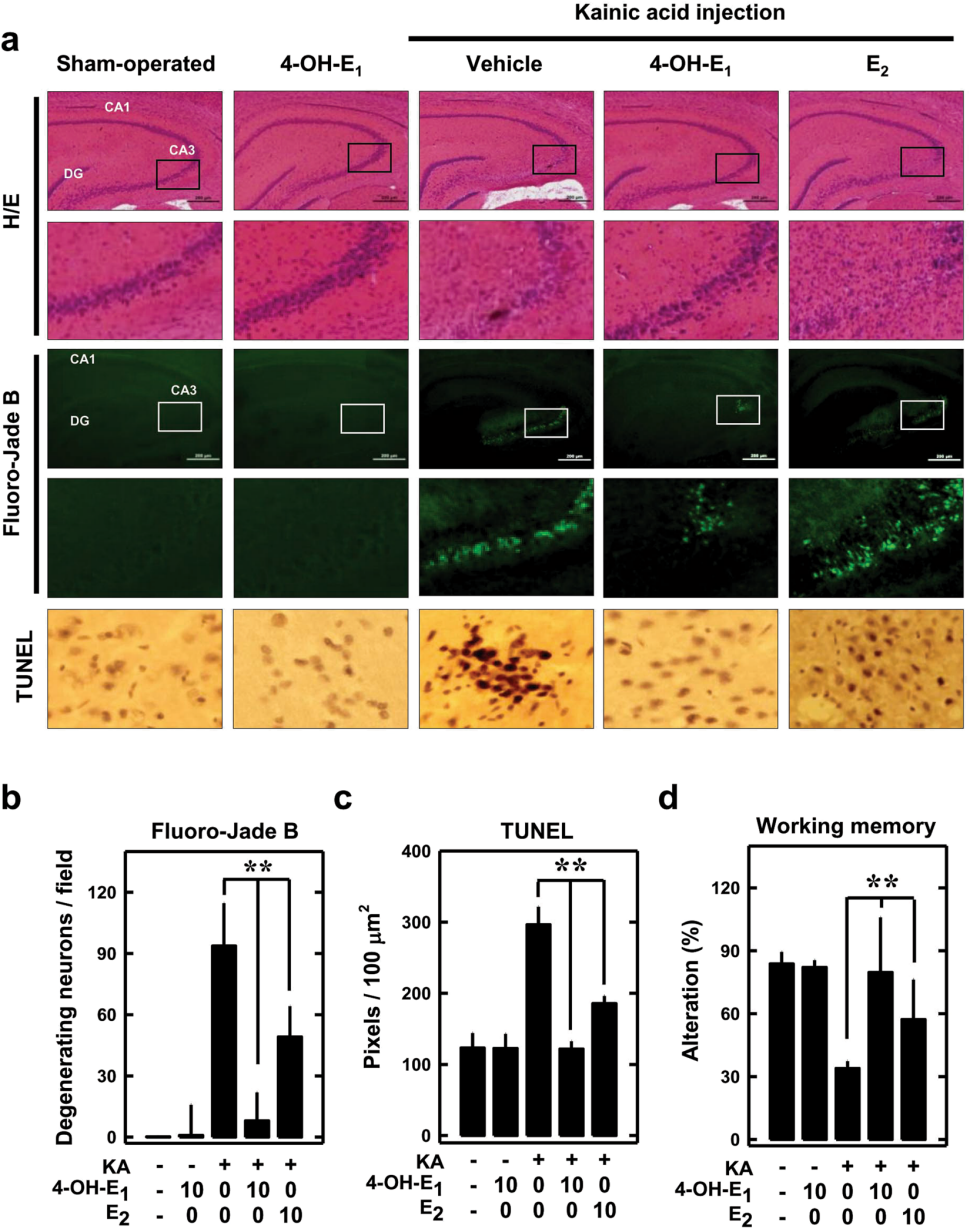
4-OH-E_1_ attenuates kainic acid (KA)-induced neurodegeneration in rat hippocampus *in vivo*. **(a)** KA (1 µL of 1 µg/µL solution) is injected into the left and right lateral ventricles of male rats (anterior/posterior, ‒1.0; rostral, ±1.6; dorsal/ventral, 4.5) using a microliter syringe under anesthesia with ketamine and xylazine (50 and 5 mg/kg, *s.c*.). The control rats (sham-operated) are injected with 1 µL of saline. Immediately following kainic acid injection, the animals receive daily *s.c*. administration of 10 µg/rat of 4-OH-E_1_ or E_2_ for 7 consecutive days. Control rats receive the same *s.c*. administrations of vehicle only. Shown are representative data for the histopathological analysis (H/E staining) of the hippocampal region (H/E, upper panels, 40×) and the enlarged CA_3_ region (H/E, lower panels); the Fluoro-Jade B staining of the hippocampal region (Fluoro-Jade B, upper panels) and the enlarged CA_3_ region (Fluro-Jade B, lower panels); and the TUNEL staining of apoptotic cells (TUNEL). **(b,c)** Quantitative data for the number of degenerating neurons **(b)** and TUNEL-positive cells **(c)** in control and kainic acid-treated rats. **(d)** Working memory in control and kanic acid-treated rats based on the Y-maze test (conducted 6 days after kainic acid treatment). For all quantitative data, each value is the mean ± SD (n = 5). *P < 0.05 versus the KA alone group.

To determine whether 4-OH-E_1_ has neuroprotective effect *in vivo*, we use the kainic acid (KA)-induced hippocampal oxidative damage in rats as an *in vivo* model. Recently, we have shown that the JNK1-p53-GADD45α signaling cascade is similarly activated both in cultured HT22 neuronal cells following glutamate treatment and in rat CA3 hippocampal neurons *in vivo* following KA injection^40^. Hence, the KA-induced hippocampal injury model is considered a suitable *in vivo* model for testing the protective effect of estrogens against oxidative neuronal damage. Histological analysis of the CA3 hippocampal region at 7 days post intracerebroventricular injection of KA shows drastic neuronal loss, but co-treatment with 4-OH-E_1_ (at a very low dose of only 10 µg/rat) for 7 days affords almost complete neuroprotection in this brain region (Fig. 2a). In comparison, E_2_ at the same dose is markedly less effective. The extent of neurodegeneration in hippocampus is assessed using different methods (H/E, Fluoro-Jade B, and TUNEL stainings), and similar findings are made (Fig. 2a–c). Functional analysis shows that rats treated with KA has a significant decrease in working memory compared to control animals (*P* < 0.05; Fig. 2d), and this decrease is strongly protected by co-treatment with 4-OH-E_1_ (*P* < 0.05). Again, E_2_ had a weaker effect (Fig. 2d).

### 4-OH-E_1_ induces cytoplasmic p53 translocation

**Figure 3:**
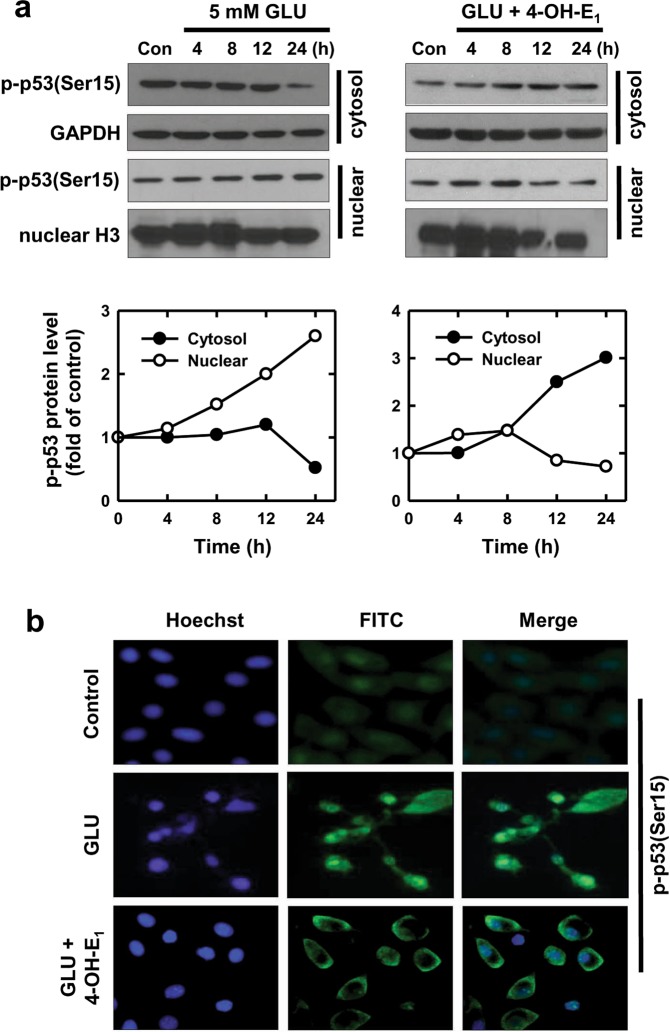
Effect of 4-OH-E_1_ on glutamate (GLU)-induced changes in p-p53(Ser15) subcellular localization in HT22 hippocampal neuronal cells. **(a)** Cells are treated with 5 mM glutamate alone or in the co-presence of 4-OH-E_1_ (5 µM) for the indicated length of time. Cytosolic and nuclear extracts are prepared from these cells, and the p-p53(Ser15) protein is analyzed using Western blotting (upper panel). The lower panels show the relative p-p53(Ser15) levels that are determined by densitometric scanning of the blots and normalized according to GAPDH (an internal control for cytoplasmic fraction) and histone H3 (an internal control for the nuclear fraction). This experiment was repeated a few times, and similar trends were observed. The data shown here is a representative data set. **(b)** Immunofluorescence microscopy analysis of the subcellular localization of p-p53(Ser15) in neuronal cells treated with glutamate alone or in combination with 4-OH-E_1_.

Our earlier study revealed that p53 activation and its downstream signaling cascade play a key role in mediating the oxidative cytotoxicity in hippocampal neurons *in vitro* and also in rat CA3 hippocampal neurons *in vivo*^37^. In the present study, we seek to determine whether 4-OH-E_1_ can protect against glutamate-induced oxidative neuronal death in cultured HT22 neuronal cells by altering p53 function. As shown in Fig. 3a (left part), after treatment with glutamate alone, there is a time-dependent increase in the nuclear p-p53(Ser15) protein level, but the change in its cytosolic protein level is opposite, suggesting that there is an increased nuclear translocation of the cytoplasmic p-p53(Ser). By contrast, when cells are co-treated with 4-OH-E_1_ and glutamate, changes in the nuclear and cytoplasmic p-p53(Ser) levels are opposite to what is observed when cells are treated with glutamate alone (Fig. 3a, right part), *i.e*., there is a time-dependent increase in cytoplasmic p-p53(Ser), whereas the nuclear p-p53(Ser) level is not increased but decreased. Additional analysis using imunofluorescence microscopy confirms that over 80% of glutamate-treated cells display p-p53(Ser) nuclear staining at 24 h, but only <10% of the cells treated with 4-OH-E_1_ + glutamate show p-p53(Ser) nuclear staining (Fig. 3b). Similar cytoplasmic p53 translocation is also observed in mouse hippocampal neurons in primary culture following treatment with glutamate (Supplementary Fig. 4).

**Figure 4:**
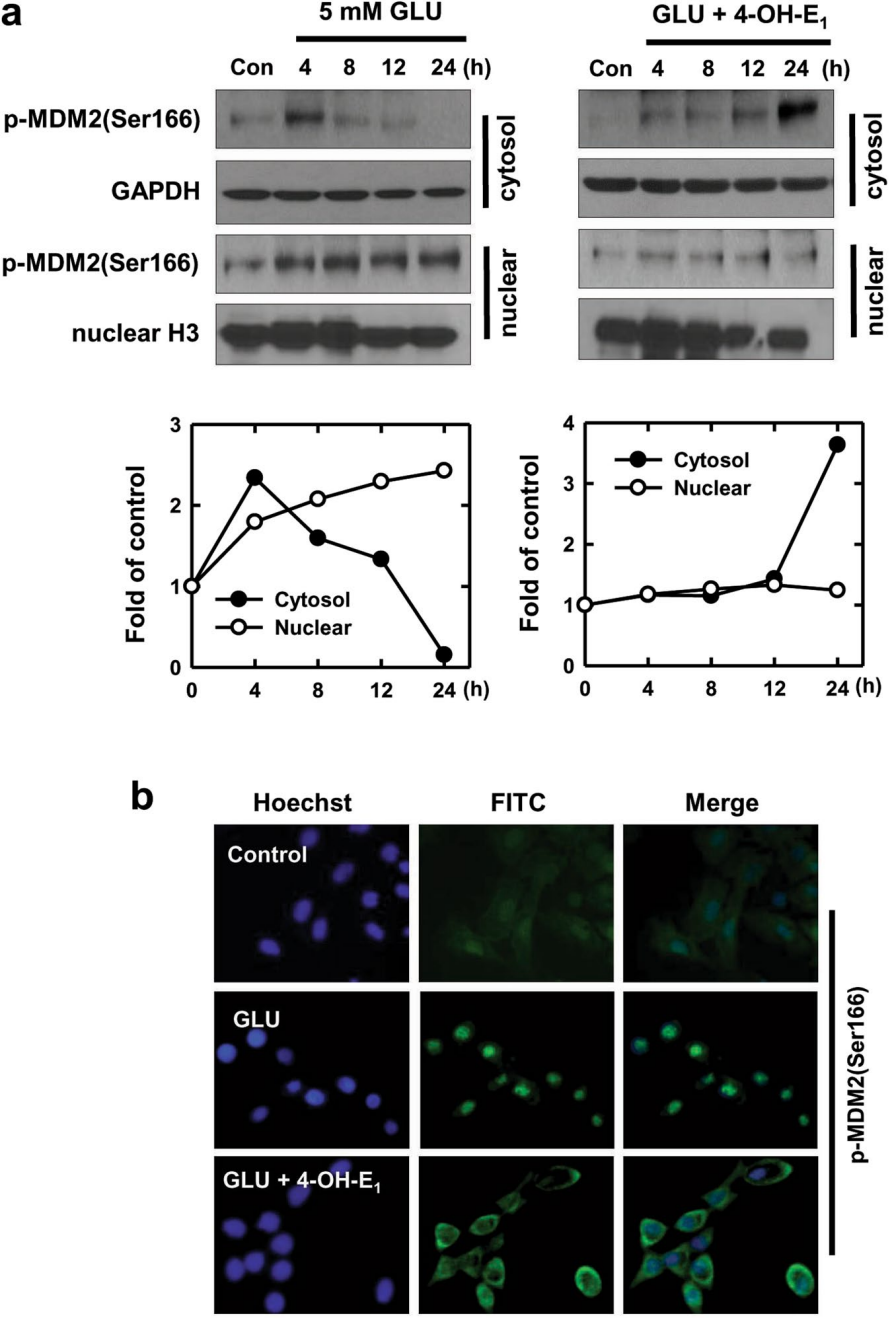
Effect of 4-OH-E_1_ on glutamate (GLU)-induced changes in p-MDM2(Ser166) subcellular localization in HT22 hippocampal neuronal cells. **(a)** Cells are treated with 5 mM glutamate alone or in the co-presence of 4-OH-E_1_ (5 µM) for the indicated length of time. Cytosolic and nuclear extracts are prepared from these cells, and the p-DM2(Ser166) protein is analyzed using Western blotting (upper panel). The lower panels show the relative p-MDM2(Ser166) levels that are determined by densitometric scanning of the blots and normalized according to GAPDH (an internal control for cytoplasmic fraction) and histone H3 (an internal control for the nuclear fraction). Note that the data obtained for this experiment used the same cellular protein extracts as used in Fig. 3. Also, this experiment was repeated a few times, and similar trends were observed. The data shown here is a representative data set. **(b)** Immunofluorescence microscopy analysis of the subcellular localization of p-MDM2(Ser166) in cells treated with glutamate alone or in combination with 4-OH-E_1_.

MDM2, an ubiquitin ligase for p53, plays a central role in regulating the stability of p53^41^. Phosphorylation of MDM2 at Ser166 increases its ability to ubiquitinate and degrade p53^42,43^. In addition, MDM2 also inhibits p53 transcriptional activation in the nuclei^41,44^. In this study, we examine the change in phosphorylated MDM2(Ser166) (p-MDM2(Ser166)) in neuronal cells treated with glutamate alone or in combination with 4-OH-E_1_. As shown in Fig. 4a, p-MDM2(Ser16) level in the cytoplasm peaks at 4 h and then gradually decreases to undetectable level at 24 h; by contrast, its nuclear level continuously increases (Fig. 4a, left panel). Analysis of immunofluorescence microscopy shows that over 80% of the cells display nuclear staining of p-MDM2(Ser166) at 24 h after glutamate exposure (Fig. 4b). When cells are co-treated with 4-OH-E_1_ and glutamate, there is a drastic increase in cytoplasmic staining of p-MDM2(Ser166) at 24 h, and almost all cells display cytoplasmic p-MDM2(Ser166) staining (Fig. 4a, right panel; Fig. 4b). In comparison, the nuclear p-MDM2(Ser16) staining remains low and is not significantly altered (Fig. 4b).

### 4-OH-E_1_ alters p53 transcriptional activity

**Figure 5:**
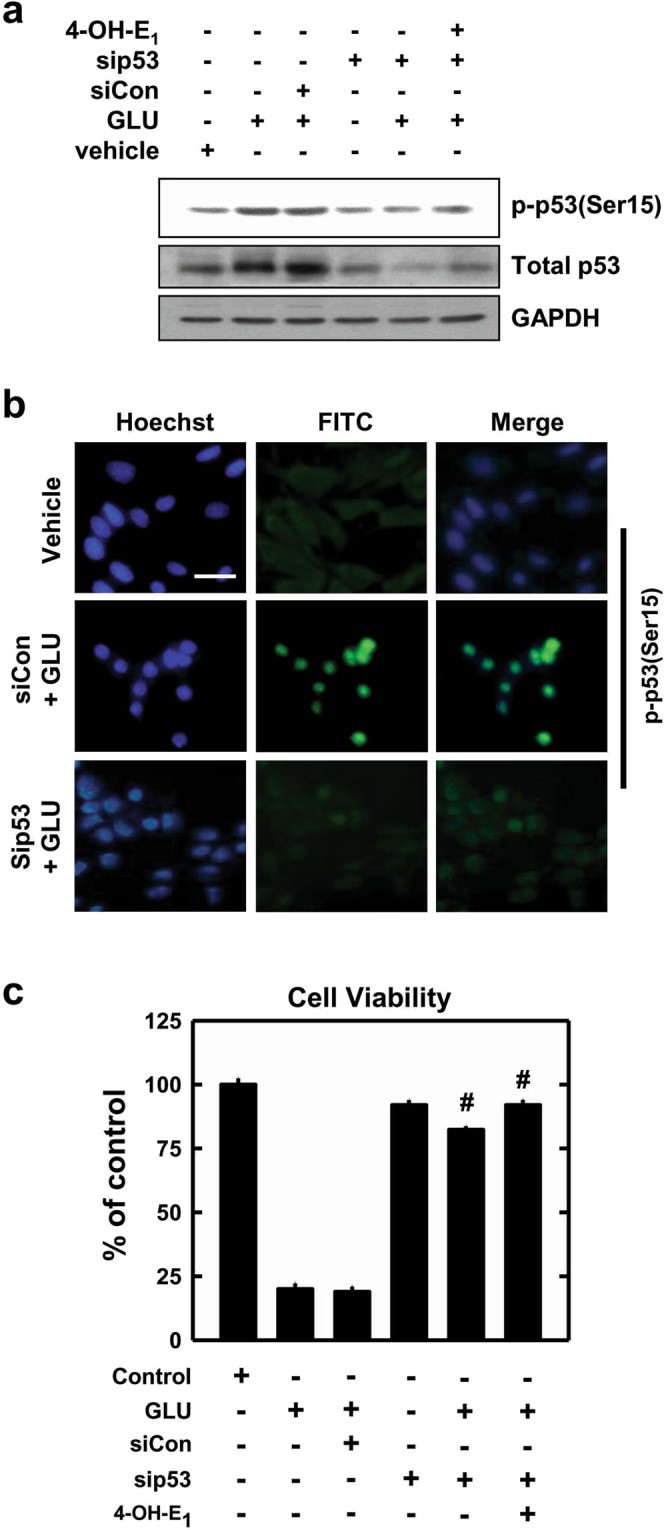
Knockdown of p53 suppresses glutamate (GLU)-induced p53 phosphorylation and cell death in HT22 hippocampal neuronal cells. **(a)** Cells are transfected with either the negative control siRNAs (siCon) or p53 siRNAs (sip53), and 24 h later, cells are exposed to 5 mM glutamate alone or in combination with 4-OH-E_1_ (5 µM) for additional 24 h. Cell lysats are subjected to Western blotting for the levels of p-p53(Ser15) and total p53 proteins. **(b)** Analysis of subcellular localization of p-p53(Ser15) by immunofluorescence staining. Cells are transfected with sip53 or siCon, and after 24 h, they are exposed to 5 mM glutamate for additional 24 h. They are then analyzed for immunofluorescence staining of p-p53(Ser15). **(c)** Changes in cell viability (based on MTT assay) in different treatment groups. Each bar is mean ± S.D. of 6‒8 replicate measurements. ^#^*P* < 0.05 versus the siCon + glutamate group.

To test the hypothesis that the observed change in p53 protein level and localization mediates the neuroprotective action of 4-OH-E_1_, the following experiments are performed. First, we use the siRNA approach to selectively knock down p53 expression. Transfection with p53 siRNA abrogates glutamate-induced increase in total p53 and p-p53(Ser15) protein level (Fig. 5a). Similarly, the glutamate-induced nuclear translocation of p53 is also mostly suppressed (Fig. 5b). These effects are correlated with a strong reduction of glutamate-induced neuronal death (Fig. 5c). In comparison, transfection with the negative control siRNAs does not have a similar effect. As expected, when neuronal cells with p53 knockdown are co-treated with glutamate and 4-OH-E_1_, the effect of 4-OH-E_1_ on p53 phosphorylation and nuclear translocation is mostly masked.

**Figure 6:**
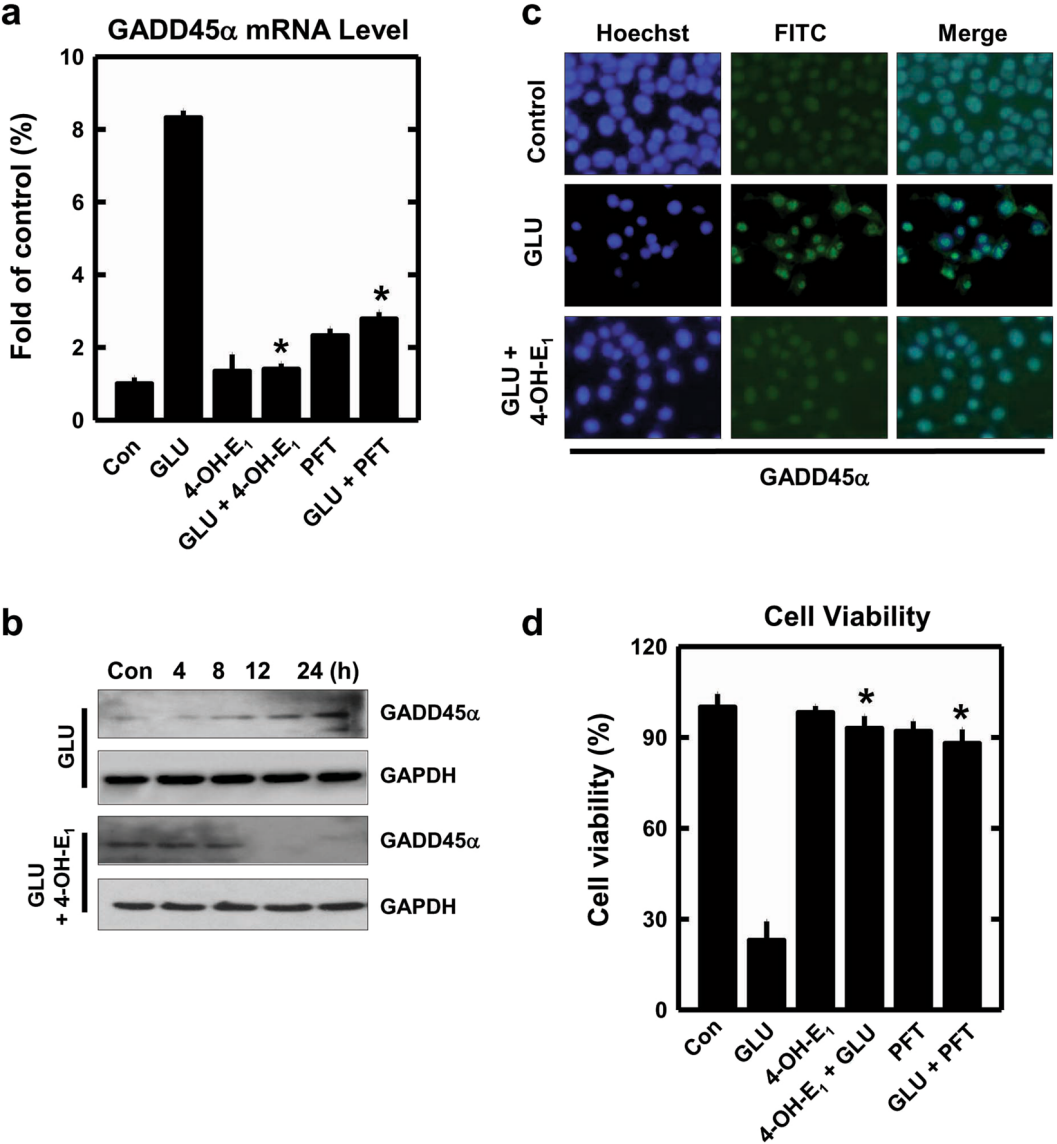
4-OH-E_1_ inhibits glutamate (GLU)-induced p53 transcriptional activity in HT22 hippocampal neuronal cells. **(a)** Cells are first pre-treated for 1 h with 10 μM PFT-α or vehicle, and then exposed to 5 mM glutamate with or without 4-OH-E_1_ (5 µM) for additional 24 h before analysis of GADD45α mRNA level by using the real-time qPCR. Each bar is a mean ± S.D. (n = 3). *P < 0.05 versus the glutamate alone group**. (b)** Cells are treated with 5 mM glutamate with or without 4-OH-E_1_ (5 μM) for the indicated length of time, and then cell lysates are subjected to Western blotting (upper panel) of GADD45α protein. **(c)** Cells receive the same treatments as in panel (b) and then are analyzed for mmunofluorescence staining of GADD45α protein in these cells. **(d)** Cells receive the same treatments as in panel (a) and then are analyzed (MTT assay) for cell viability. Each bar is mean ± S.D. (n = 6). *P < 0.05 versus the glutamate alone group.

Next, we examine whether co-treatment with 4-OH-E_1_ alters p53 transcriptional activity in neuronal cells. We determine the mRNAs of several p53-regulated genes, including GADD45α, p21, p75 and p53 (data shown in Supplementary Fig. 5). Among these genes, change in GADD45α mRNA level is most pronounced. GADD45α is identified in our recent study as a key mediator of the oxidative neuronal death, and thus it is measured as a marker of p53 transcriptional activity in this study. Its mRNA level is increased by approximately 8-fold after treatment with glutamate alone, and co-treatment with 4-OH-E_1_ almost completely abrogates GADD45α mRNA accumulation (Supplementary Fig. 5, Fig. 6a). Similarly, the induction of GADD45α mRNA by glutamate treatment is reduced by co-treatment with PFT (a known p53 inhibitor) (Fig. 6a) or by p53 knockdown^37^. Western blot analysis shows that GADD45α protein is very low in untreated cells, but is markedly elevated in a time-dependent manner in cells treated with glutamate, and co-treatment with 4-OH-E_1_ markedly reduces GADD45α protein level (Fig. 6b). Additional immunocytochemical staining of GADD45α protein shows that while this protein is basically undetectable in untreated cells, it is markedly elevated in cells treated with glutamate, and co-treatment with 4-OH-E_1_ markedly reduces GADD45α protein level (Fig. 6c). The observed changes in p53 transcriptional regulation of GADD45α expression and protein level in neuronal cells co-treated with 4-OH-E_1_ are coupled with changes in cell viability (Fig. 6d). Notably, similar changes in p53 protein and GADD45α mRNA levels are also seen in the hippocampal region of rats treated with KA, and these changes are suppressed by co-treatment with 4-OH-E_1_ or E_2_ (Supplementary Fig. 6), which is accompanied by increased neuronal survival *in vivo* (data shown in Fig. 2a). Collectively, these results show that 4-OH-E_1_ exerts its neuroprotective actions by reducing the transcriptional activity of p53 as well as its regulated gene products such as GADD45α in neuronal cells.

### Effect of 4-OH-E_1_ on SIRT1 protein level and function

**Figure 7:**
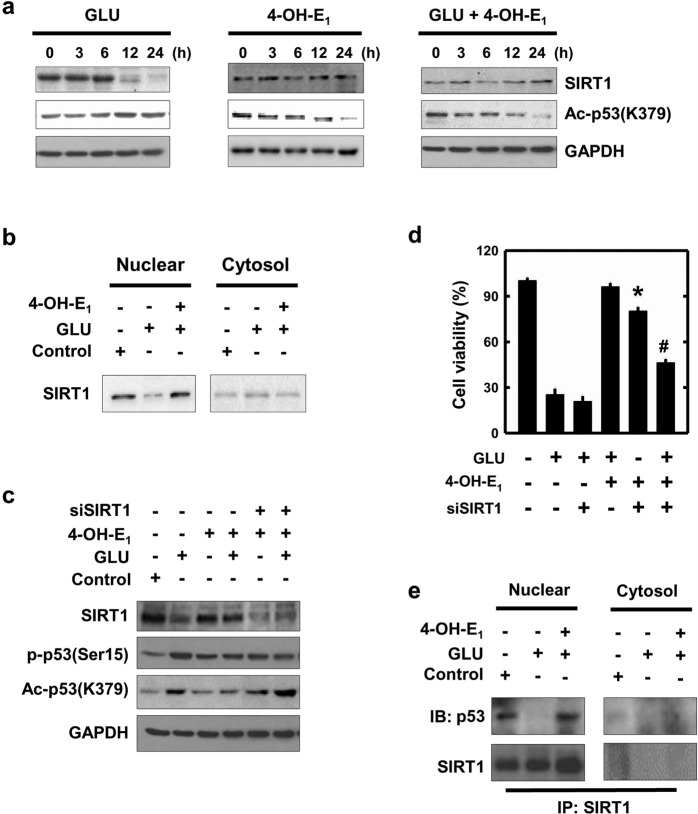
Role of SIRT1 in glutamate (GLU)-induced p53 acetylation and 4-OH-E_1_’s neuroprotection in HT22 hippocampal neuronal cells. **(a)** Cells are treated with 5 mM glutamate with or without 4-OH-E_1_ (5 µM) for 0, 3, 6, 12, and 24 h. Whole cell lysates are subjected to Western blot analysis of SIRT1 and acetyl-p53(Lys 379) levels. **(b)** Cells are treated with 5 mM glutamate with or without 4-OH-E_1_ (5 µM) for 24 h, and the cytosolic and nuclear extracts are prepared from these cells. SIRT1 protein is analyzed using Western blotting. **(c)** Cells are transfected with the SIRT1 siRNAs (siSIRT1), and 24 h later, cells are exposed to 5 mM glutamate with or without 4-OH-E_1_ (5 µM) for additional 24 h. Cell lysates are subjected to Western blotting of the levels of SIRT1, p-p53(Ser15), and acetylated-p53(Lys 379). **(d)** Cell viability is determined using the MTT assay. Note that the SIRT1 protein levels are repeated twice, and similar observations are made. **(e)** Cells are treated with 5 mM glutamate with or without 4-OH-E_1_ (5 µM) for 24 h and then co-immunoprecipitation of SIRT1 is carried out. Cytosolic and nuclear extracts are prepared from these cells, and the SIRT1 or p53 is analyzed using Western blotting.

As shown in Fig. 7a and Supplementary Fig. 7a, treatment of HT22 cells with glutamate results in a time-dependent decrease in SIRT1 protein level, and co-treatment with 4-OH-E_1_ and glutamate restores cellular SIRT1 protein level. It is known that acetylated p53 is a substrate of SIRT1^45,46^, and the acetyl-p53 is the transcriptionally-active form present in the nuclei^46^. Hence, next we choose to determine whether 4-OH-E_1_ can inhibit glutamate-induced acetylation of p53. As shown in Fig. 7a, p53 acetylation level is increased by glutamate treatment in a time-dependent manner. When cells are treated with 4-OH-E_1_ alone or in combination with glutamate, acetyl-p53 protein level is decreased at 12 and 24 h. When the nuclear and cytosolic fractions are separately analyzed for SIRT1 protein level, it is observed that SIRT1 is mostly present in the nuclear extracts from cells treated with vehicle or glutamate + 4-OH-E_1_, but its nuclear level is markedly reduced in cells treated with glutamate alone (Fig. 7b).

Next, we determine whether SIRT1 can modulate glutamate-induced p53 acetylation. The p53 acetylation level in SIRT1-knockdown HT22 cells remains high following co-treatment with glutamate and 4-OH-E_1_ (Fig. 7c, Supplementary Fig. 7b), and the extent of glutamate-induced cell death is not significantly altered by the co-treatment (Fig. 7d). Notably, SIRT1 knockdown does not affect glutamate-induced p53 phosphorylation (Fig. 7c). Using immunoprecipitation analysis, we also examine the interaction between SIRT1 and p53. As shown in Fig. 7e, p53 is detected in SIRT1-immunoprecipitated nuclear fraction, whereas p53 and SIRT1 are not detected in cytosolic fraction.

Together, these data show that SIRT1 is an important upstream target protein that mediates 4-OH-E_1_’s neuroprotective effect by deacetylating p53 and thereby causing its cytosolic translocation.

### Metabolism of E_2_ by rat brain tissue

**Figure 8:**
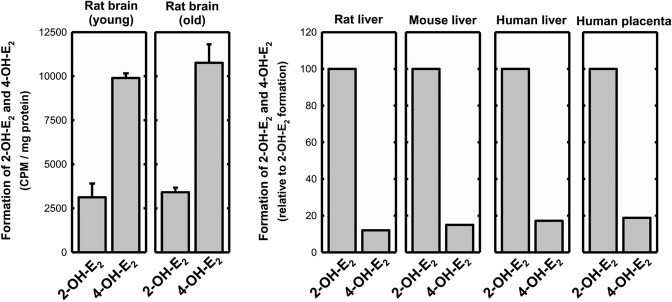
Metabolic conversion of [^3^H]E_2_ to 2-OH-E_2_ and 4-OH-E_2_ catalyzed by rat brain microsomes in the presence of NADPH as a cofactor (left panel). Each value is the mean ± S.D. from brain microsomes prepared from four groups of male rats. For comparison, the relative ratios of E_2_ 2-hydroxylation to 4-hydroxylation catalyzed by microsomes prepared from pooled rat and mouse liver tissues are also determined, with each value representing the mean of replicate measurements (right panel). In addition, we also included the average ratio of E_2_ 2- and 4-hydroxylation by human liver and placental microsomes (right panel), which are taken from our earlier studies^49–51^ and are included here for ease of comparison.

To test the hypothesis that certain estrogen metabolites are metabolically formed in the brain to serve as neuroactive estrogens, we determine the estrogen metabolite profiles when [^3^H]E_2_ is incubated with rat brain microsomes (which contain various estrogen-metabolizing cytochrome P450 enzymes). As shown in Fig. 8, the ratio of E_2_ 4-hydroxylation to 2-hydroxylation by rat brain microsomes is distinctly different from the ratios observed with rat and mouse liver microsomes under the same incubation conditions. Earlier studies have shown that 2-OH-E_2_ and 2-OH-E_1_ are quantitatively-major metabolites formed with rat, mouse or human liver microsomes or with human placental microsomes when [^3^H]E_2_ and/or [^3^H]E_1_, respectively, are used as substrates^47–51^. The predominance of E_2_ 2-hydoxylation over 4-hydroxylation by rat or mouse liver microsomes (by an average ratio of 6.5 to 1) is also observed in the present study (Fig. 8, right panel), and these data are also very consistent with earlier reports^47,48^. However, when rat brain microsomes are used as the enzyme source, 4-OH-E_2_ becomes the more predominant metabolite of E_2_ over 2-OH-E_2_, with an average ratio of 4 to 1 (Fig. 8, left panel). It is of note that our earlier study showed that among the 15 commonly-known CYP isoforms tested, CYP1B1 (which is known to be expressed in the brain^52,53^) is the only CYP with a preferential estrogen 4-hydroxylase activity, forming 4-OH-E_2_ or 4-OH-E_1_ at a ratio of 3~4 to 1 over 2-OH-E_2_ or 2-OH-E_1_^44^. These data suggest that 4-OH-estrogens are quantitatively predominant estrogen metabolites of E_2_ and E_1_ formed in the brain, and their formation likely is catalyzed by CYP1B1.

## Discussion

The present study seeks to investigate the protective effect of endogenous estrogen metabolites against oxidative neurotoxicity using both *in vitro* and *in vivo* models, and also to determine the mechanism of their neuroprotective action. Among a group of 25 endogenous E_2_ and E_1_ metabolites tested, we find that 4-OH-E_1_, an endogenous metabolite of E_1_ formed by cytochrome P450 enzymes, has the strongest neuroprotective effect against oxidative neurotoxicity in HT22 hippocampal neuronal cells *in vitro*. Its protective effect is also markedly stronger than its parent hormones E_1_ and E_2_. Similarly, 4-OH-E_1_ is found to have a markedly stronger effect than E_2_ in protecting against hippocampal oxidative damage in rats treated with kainic acid. We show that estrogen 4-hydroxylation is the main metabolic pathway of endogenous estrogens catalyzed by brain microsomes which contain all cellular cytochrome P450 enzymes, suggesting that 4-hydroxyestrogens are the main estrogen metabolites formed in the CNS.

It is of note that the neuroprotective effect of 4-OH-E_1_ in male rats is achieved when the animals are given an *i.p*. injection of only 10 µg/rat. Earlier studies using ovariectomized female rats show that the dose-response curve for the uterotropic effect of E_1_ is in the dose range of 1‒50 µg/rat (*i.p*. or *i.m*. injection)^54,55^. The dose of 10 µg/rat of E_2_ or E_1_ would yield 50‒70% of the maximal uterotropic activity in ovariectomized female rats, suggesting that this neuroprotective dose of endogenous estrogens is physiologically relevant.

Mechanistically, the results of this study show that 4-OH-E_1_ attenuates glutamate-induced neuronal death by accumulating p-p53(Ser15) in cytoplasm. Moreover, induction of GADD45α in HT22 cells following glutamate-induced oxidative cytotoxicity is dependent on the nuclear transcriptional function of p-p53(Ser15). Our recent study showed that selective knockdown of p53 expression or use of a p53 inhibitor, PFT-α, each can attenuate glutamate-induced GADD45α mRNA and protein level in HT22 cells, which is accompanied by a strong protection against glutamate-induced neuronal death^37^.

MDM2, particularly its phosphorylated form p-MDM2(Ser166), plays a central role in regulating the stability of p53^41–44,56–58^. In addition, MDM2 can bind to the transactivation domain of p53 and thereby interfere with its recruitment of basal transcription machinery. We find that p-MDM2(Ser166) is translocated into the nuclei following glutamate treatment. However, when the cells are co-treated with 4-OH-E_1_, there is an increased cytoplasmic localization, in a similar manner as p53. Collectively, these data suggest that the increased cytoplasmic retention of p53 and MDM2 plays a role in mediating the protective action of 4-OH-E_1_ in glutamate-treated HT22 neuronal cells.

It is known that the level of acetyl-p53 is down-regulated by SIRT1, which then inhibits the p53-dependent cell death^59^. In this study, we find that treatment with glutamate alone results in a strong down-regulation of SIRT1 protein level, accompanied by nuclear accumulation of acetyl-p53 protein. In addition, co-treatment of these cells with 4-OH-E_1_ prevents the glutamate-induced reduction in SIRT1 protein level. Since SIRT1 knockdown is shown to increase the nuclear level of acetyl-p53 and abolish the protective action of 4-OH-E_1_ against glutamate neurotoxicity, these findings suggest that 4-OH-E_1_ exerts its protection by promoting SIRT1-mediated p53 deacetylation, which then facilitates p53 cytoplasmic translocation and thereby suppresses p53-dependent neuronal death.

Earlier studies show that the presence of a free phenolic 3-OH group on the steroidal estrogens is critical for their neuroprotective effect^8^. Notably, all the estrogen metabolites tested in this study contain a free phenolic 3-OH group, but their neurprotective effect is vastly different. Since some of the estrogen metabolites do not have a neuroprotective effect, this observation suggests that the presence of phenolic group(s) is not sufficient. This observation also rules out the predominance of the simple chemical antioxidant activity (chemical reductivity) of their phenolic moiety as the chief mechanism of neuroprotective actions. In further support of the notion that the neuroprotective effect of 4-OH-E_1_ is not due to its antioxidant activity, it should be noted that 4-OH-E_2_ is actually a well-known pro-oxidant (reviewed in ref. ^60,61^). In agreement with this suggestion, *N*-acetylcysteine, an antioxidant that can strongly protect against glutamate-induced neuronal death, does not have a similar effect as 4-OH-E_1_ to induce cytoplasmic p53 translocation. Together, these findings suggest that 4-OH-E_1_ can selectively promote p53 cytoplasmic translocation, which results from SIRT1-mediated p53 deacetylation.

At present, it remains controversial regarding the role of estrogen receptor (ER)-dependent transcription in mediating estrogen’s neuroprotective actions. It has been reported that 17α-E_2_, an estrogen that can only weakly activate ER-dependent gene transcription, has an equipotent neuroprotective efficacy as E_2_^12^. In the mouse model of cerebral ischemia, the data are equally inconclusive regarding the role of ERs in mediating the neuroprotective effects^62^. Since 4-OH-E_1_ has little or no binding affinity for ERα and ERβ^45^, it provides support for the suggestion that the neuroprotective effect of aromatic estrogens is partly unrelated to the ERs.

In summary, the mechanism of 4-OH-E_1_′s neuroprotective effect has two main elements: (**1**) 4-OH-E_1_ protects neuronal survival by altering the function of p53 through promoting its translocation from nucleus to cytoplasm, thereby reducing its transcriptional activity and the expression of its downstream target genes (including GADD45α). (**2**) The observed cytoplasmic translocation of p53 during co-treatment with 4-OH-E_1_ is due to the maintenance of the SIRT1 protein level and ultimately its function in glutamate-treated neuronal cells. Because SIRT1 is a protein deacetylase, the maintenance of its function would maintain the basal level of p53 deacetylation in the nucleus, which then promotes p53 nuclear exit and neuronal survival.

## Materials and Methods

### Chemicals

*l*-Glutamic acid, estrogens and their metabolic derivatives, and fetal bovine serum (FBS) are obtained from Sigma (St. Louis, MO). Dullbecco’s modified Eagle’s medium (DMEM) is obtained from Life Technology (Rockville, MD). The antibiotics solution (containing 10,000 U/mL penicillin and 10 mg/mL streptomycin) is obtained from Invitrogen (Carlsbad, CA), and trypsin-versene mixture (containing 0.25% trypsin and 0.02% EDTA) from Lonza Walkersville (Walkersville, MD). Pifithrin-α (PFT-α, p53 inhibitor) is obtained from Calbiochem (San Diego, CA).

### Neuronal cell culture conditions and assay of cell viability

The HT22 cells (immortalized mouse hippocampal cell line) are a gift from Dr. David Schubert (Salk Institute, La Jolla, CA), and The cells are maintained in the DMEM supplemented with 10% (*v/v*) FBS and antibiotics (penicillin-streptomycin), and incubated at 37 °C under 5% CO_2_^36–38^.

Cell viability is measured using the MTT assay. Cells are seeded in 96-well plates (5 × 10^3^ cells/well), and treated with glutamate for 24 h. 10 μL MTT (at 5 mg/mL) is added to each well, and the cells are incubated for 3 h. Dimethyl sulfoxide (DMSO, 100 μL) is then added to each well, and the absorbance is read with a UV max microplate reader (Molecular Device, Palo Alto, CA) at 560 nm. The relative cell viability is expressed as a percentage of the control cells that were not treated with glutamate.

### Immunofluorescence microscopy

Cells are first fixed with 3% paraformaldehyde solution for 10 min. Cells are then permeabilized in 0.2% Triton® X-100/PBS for 5 min, and blocked with 10% normal goat serum (Jackson Immuno Research Labs, West Grove, PA) for 1 h. GADD45α monoclonal antibody (1:75; Santa Cruz, CA) and p-p53(Ser15) polyclonal antibody (1:100; Cell Signaling Technology), are added to the slides and the slides were incubated for 24 h at 4 °C. Fluorescein isothiocyanate (FITC)-conjugated secondary antibody (1:200; Jackson Immuno Research Labs) is then added. The nuclei are stained with Hoechst33342, and the coverslips are mounted on slides with Vectashield Mounting Medium (Vector Larboratories, Burlingame, CA). Fluorescein images are captured using a confocal fluorescence microscope (AXIO, Carl Zeiss Corporation, Germany).

### TUNEL assay

The ApopTag plus peroxidase *in situ* apoptosis detection kit (Chemicon, Temecula, CA) is used to detect TUNEL-positive cells. Cells are stained according to the manufacturer’s instructions. *In situ*-labeled nuclei are observed and photographed under a fluorescence microscope (AXIO, Carl Zeiss).

### Flow cytometric analysis

Cells are stained with 50 μg/mL propidium iodide (PI) for analysis of cell cycles or annexin-V and PI using the annexin-V-FITC apoptosis detection kit (BD Bioscience, San Jose, CA) for analysis of apoptosis. For cell cycle analysis, cells are resuspended in 1 mL of 0.9% NaCl, and 2.5 mL of ice-cold 90% ethanol are added. After incubation at room temperature for 30 min, cells are centrifuged and the supernatant is removed. Cells are resuspended in 1 mL PBS containing PI and 100 μg/mL ribonuclease A and incubated at 37 °C for 30 min. After centrifugation, cells are resuspended in PBS. For annexin V-PI double staining, the procedure is performed according to manufacturers’ protocols. Flow cytometric analyses are performed by using a flow cytometer (model BD LSR II, BD Bioscience, San Jose, CA).

### Nuclear and cytoplasm extracts

For protein localization, the nuclear and cytosolic fractions are prepared using the cytosolic/nuclear fractionation kit obtained from Biovision Inc. (Mountain View, CA), by following the instructions of the manufacturer. Briefly, cells are suspended in hypotonic buffer and lysed with the proprietary detergent from the kit. Samples are spun at 800 g for 10 min at 4 °C. The supernatant is collected, spun 5 min at 16,000 g to remove any remaining nuclei, and then transferred to a new microtube (cytosolic protein fraction). The original pellet is resuspended in the nuclear extraction buffer and then incubated on ice for 40 min with occasional vortexing. After salt extraction, the nuclear pellet is centrifuged at 16,000 g for 10 min, and the supernatant is saved as the nuclear extract.

### Western blotting

Cells are washed first, and then suspended in 100 μL of RIPA buffer (Sigma) and a protease inhibitor cocktail. The amount of proteins is determined using the Bio-Rad protein assay (Bio-Rad, Hercules, CA). The proteins are separated by 10% SDS-polyacrylamide gel electrophoresis (SDS-PAGE) and electrically transferred to a polyvinylidene difluoride membrane (Bio-Rad). After the membrane is blocked using 5% skim milk, target proteins are immunodetected using specific antibodies. Thereafter, the horseradish peroxidase (HRP)-conjugated anti-rabbit IgG is applied as the secondary antibody, and the positive bands are detected using Amersham ECL plus Western blotting detection reagents (GE Health care, Piscataway, NJ).

### Small-interfering RNA (siRNA)

The role of GADD45α and p53 in mediating glutamate oxidative cytotoxicity is examined using GADD45α-siRNA (siGADD45α) and p53-siRNA (sip53) to selectively silence the GADD45α and p53, respectively. The siGADD45α (catalog no. sc-35439, Santa Cruz, CA), sip53 (catalog no. sc-29436, Santa Cruz), and siRNA negative control (catalog no. sc-37007, Santa Cruz) are purchased from Santa Cruz Biotechnology. HT22 cells are seeded the night before transfection at a density of 30‒50% confluence by the time of transfection. Forty nmol of siGADD45α, sip53, and siRNA negative control are used for transfection using Lipofectamine 2000 (Invitrogen, San Diego, CA) according to the manufacturer’s instructions. Transfected cells are maintained in culture for 2 days before harvesting and further analyses. The efficiency of the siRNA knockdown is determined by Western blot analysis.

### RNA isolation and cDNA synthesis

Total RNA is isolated using TRIzol (Invitrogen) from HT22 cells treated with 5 mM glutamate. cDNA is synthesized with random hexamers (PerkinElmer Life Sciences, Boston, MA, and Roche Applied Science, Indianapolis, IN) using Moloney murine leukemia virus-reverse transcriptase (PerkinElmer Life Sciences).

### Real-time PCR

Real-time PCR is performed on a PE Biosystems ABI PRISM 7300 sequence detection system. In brief, total RNA is isolated from cultured cells using the RNeasy^®^ Mini Kit (Qiagen, Venlo, Netherlands) according to the manufacturer’s procedure. Quantitative RT-PCR is done with a CYBR green RT-PCR kit (Applied Biosystems, Foster City, CA). A 25-μL reaction contained 1× CYBR green reaction mix with 50 ng of primers and 1 μL cDNA from the reverse transcription. Forty cycles of PCR reactions are done with 30 seconds of denaturing at 95 °C, 30 seconds of annealing at 60 °C, and 1 min of PCR reaction at 72 °C. The primers for GADD45α are: forward (5′-CTGGAGGAAGTGCTCAGCAAGG-3′), reverse (5′-CTGATCCATGTA GCGACTTTCC-3′), and the primers for GAPDH: forward (5′-GGCACAGTCAAGGCCGAGAA-3′), reverse (5′-CAGCAATGCATCCTGCACCA-3′). Fold of induction of the expression is determined according to a published method^63^. Analysis of relative gene expression data using real-time quantitative PCR and the 2(-ΔΔC(T)) method. Briefly, the method involves obtaining the C_T_ values for the GADD45α mRNA, normalizing to GAPDH (a house-keeping gene), and then deriving the fold of increase compared to the corresponding control cells.

### *In vivo* experiments animal treatment

All procedures involving the use of live animals as described in this study are approved by the Institutional Animal Care and Use Committee (IACUC) at the University of Kansas Medical Center (KUMC), and the guidelines for humane care of animals set forth by the National Institutes of Health are followed. Male Sprague-Dawley rats, weighing 250–270 g, are purchased from Harlan (Indianapolis, IN). After arrival, the animals are allowed to acclimatize to the new environment for one week before used in the experiment. Rats are randomly divided into various experimental groups (n = 5) with no significant difference in average body weight.

Kainic acid (1 µL of 1 µg/µL solution) is injected into the left and right lateral ventricles (anterior/posterior, ‒1.0; rostral, ±1.6; dorsal/ventral, 4.5) using a microliter syringe under anesthesia with ketamine and xylazine (50 and 5 mg/kg, *s.c*.). The control rats are injected with 1 µL of saline instead of kainic acid^64^. The animals are administered *s.c*. 10 µg/rat of E_2_ or 4-OH-E_1_ for 7 consecutive days. This protocol produces E_2_ levels of ~30 pg/mL at 24 h post injection^65^. Control rats received *s.c*. administrations of vehicle only.

### Y-Maze test

The degree of memory impairments in rats is measured using the Y-maze test^66^ after six days of administration. The Y-maze is a three-arm maze with equal angles between all arms, which are 50 cm in length and 10 cm in width, with walls 20-cm high. The maze floor and walls are constructed from dark grey polyvinyl plastic. Rats are initially placed within one arm, and the sequence and number of arm entries are recorded manually. The percentage of triads with all three arms represented, *i.e*., ABC, CAB, or BCA, but not ABB, is recorded as an alternation to estimate the short-term memory. Arms are cleaned by using 70% ethanol between tests to remove odors and residues. The alternation score (%) for each rat is defined as the ratio of the actual number of alternations to the possible number (defined as the total number of arm entries minus two) multiplied by 100 as shown by the following equation:

$$ \% \,{\rm{alternation}}=[({\rm{number}}\,{\rm{of}}\,{\rm{alternations}})/({\rm{total}}\,{\rm{arm}}\,{\rm{entries}}-2)]\times 100.$$

### Immunohistochemistry staining

At the end of the experiment before collecting the brain tissue for analysis, the animals receive ketamine and xylazine (50 and 5 mg/kg, *s.c*.) for anesthesia, and then is perfused with physiological saline (0.9% NaCl) via the abdominal aorta. The collected brain tissues are postfixed overnight in the same fixative solution. After cryoprotection in 30% sucrose/phosphate buffer, the tissues are frozen in liquid nitrogen and sectioned serially (30 µm) through the entire brain. The sections are collected in 0.1 M neutral phosphate buffer, mounted on slides, then air-dried on a slide warmer at 50 °C for at least half an h, and stained with hematoxylin and eosin (H/E) for histopathological analysis. Three brain hippocampal regions (CA1, CA3, and the dentate gyrus DG) at the level of bregma ‒3.6 mm are examined bilaterally^67^.

Similarly, to detect the apoptotic DNA degradation in brain tissue slides, we use the terminal deoxynucleotidyl transferase (TdT)-mediated dUDP-biotin nick end labeling (TUNEL) method, *i.e*., the ApopTag^®^ Plus Peroxidase *In Situ* Apoptosis Detection Kit (Chemicon International, Temecula, CA).

Fluoro-Jade B staining is performed by following the protocols described by Schmued and Hopkins^68^ with minor modifications. Briefly, the slides are transferred to a solution of 0.06% potassium permanganate for 10 min, preferably on a shaker table to insure consistent background suppression between sections. The staining solution is prepared from a 0.01% stock solution of Fluoro-Jade B that is made by adding 10 mg of the dye powder to 100 mL of distilled water. After 20 min in the staining solution, the slides are rinsed and placed on a slide warmer until they are fully dry. Cell counts are performed using grid morphometric techniques under low- and high- power magnification fields.

### *In vitro* metabolism of [^3^H]E_2_ by rat brain microsomes

Brains from 16 rats (eight 4-week-old young rats and eight 16-week-old rats) are collected. Two brains from the same age group of rats are randomly pooled for preparation of brain microsomes. Rat and mouse liver microsomes are prepared from pooled liver tissues. For the *in vitro* metabolism, [^3^H]E_2_ is used as a substrate, and incubated with the rat brain or liver microsomes in the presence of ascorbic acid and NADPH as described earlier^69,70^. Briefly, the incubation mixture consists of 1 mg/mL microsomal protein, 1 μM [^3^H]E_2_ as substrate (containing 3 μCi [^3^H]E_2_, specificity 110 Ci/mmol), 2 mM NADPH as cofactor, and 2 mM ascorbic acid as a reducing agent in the final volume of 250 μL of 0.1 M phosphate buffer (pH 7.4). The *in vitro* metabolism lasts for 1 h at 37 °C with constant mild shaking.

Here it should also be noted that all the glass tubes used in the metabolism study are pre-silanized with 5% (*v/v*) dimethyldichlorosilane in toluene for 10 min, and followed by sequential rinses in pure toluene, pure methanol, and distilled water^58,59^. The benefits of using the pre-silanized glass tubes in quantifying very small amounts of metabolically-formed catechol estrogens were addressed in detail in our earlier studies^69,70^.

Analysis of [^3^H]E_2_ metabolites is performed with an HPLC system coupled with in-line UV and radioactivity detections as described earlier^69,70^. The HPLC system consists of a Waters 2690 separation module, a Waters UV detector (model 484), an IN/US β-RAM radioactivity detector, and an Ultracarb 5 ODS column (150 × 4.60 mm, Phenomenex, Torrance, CA). The solvent system for separation of E_2_ and its metabolites consist of acetonitrile (solvent A), 0.1% acetic acid in water (solvent B), and 0.1% acetic acid in methanol (solvent C). The solvent gradient (A/B/C) used for eluting estrogen metabolites is as follows: 8 min of isocratic at an initial composition of 16/68/16, 7 min of a concave gradient (curve number 9) to 18/64/18, 13 min of a concave gradient (curve number 8) to 20/59/21, 10 min of a convex gradient (curve number 2) to 22/57/21, 13 min of a concave gradient (curve number 8) to 58/21/21, followed by a 0.1-min step to 92/5/3 and a 8.9-min isocratic period at 92/5/3. The gradient is then returned to the initial composition (16/68/16) and held for 10 min before analysis of the next sample.

The HPLC retention times for the authentic estrogen metabolites are determined by using in-line UV detection, whereas the [^3^H]E_2_ metabolite peaks formed with rat brain and liver microsomes are determined by using in-line radioactivity detection. The calculation of the amount of each estrogen metabolite formed is based on the amount of radioactivity detected for each corresponding metabolite peak, as described in our earlier studies^69,70^.

### Statistical analysis

Most of the quantitative data are expressed as mean ± S.D. Comparisons between two groups are analyzed using *ANOVA*, and a *P* value of <0.05 are considered statistically significant.

## Supplementary information

Supplementary information
