# Supplementary material for: 4-Hydroxyestrone, an Endogenous Estrogen Metabolite, Can Strongly Protect Neuronal Cells Against Oxidative Damage
Source: Sci Rep. 2020 Apr 29;10:7283. doi: 10.1038/s41598-020-62984-y (PMC7190733; doi:10.1038/s41598-020-62984-y)
Supplement: Supplementary file 1 — Supplementary information [file 41598_2020_62984_MOESM1_ESM.pdf]

4-Hydroxyestrone, an Endogenous Estrogen Metabolite, Can Strongly Protect Neuronal Cells Against Oxidative Damage

Hye Joung Choi, Anthony J. Lee, Ki Sung Kang, Ji Hoon Song and Bao Ting Zhu

School of Life and Health Sciences, The Chinese University of Hong Kong, Shenzhen, Guangdong 518172, China (H.J.C., A.J.L., B.T.Z.), and
Department of Pharmacology, Toxicology and Therapeutics, School of Medicine, University of Kansas Medical Center, Kansas City, KS 66160, USA (H.J.C., K.S.K., J.H.S., B.T.Z.)

Running title: **Effect of Endogenous Neuroprotective Estrogens**

* **Correspondence:** Bao Ting Zhu, School of Life and Health Sciences, The Chinese University of Hong Kong, 2001 Longxiang Blvd, Longgang District, Shenzhen, Guangdong, China 518172.
E-mail: **BTZhu@CUHK.edu.cn**. Phone: **+086-755-84273851**.

CAPSULE

Background: This study investigates the protective effect of endogenous estrogen metabolites against oxidative neurotoxicity and the mechanism of neuroprotection.

Results: 4-Hydroxyestrone has a strong protective effect against oxidative neurotoxicity *in vitro* and *in vivo* by promoting p53 cytoplasmic localization. 4-Hydroxyestrone is a major metabolite formed by brain microsomal enzymes.

Conclusion: 4-Hydroxyestrone is an endogenous neuroprotective estrogen.

Significance: 4-Hydroxyestrone and analogs may be developed as neuroprotective agents.

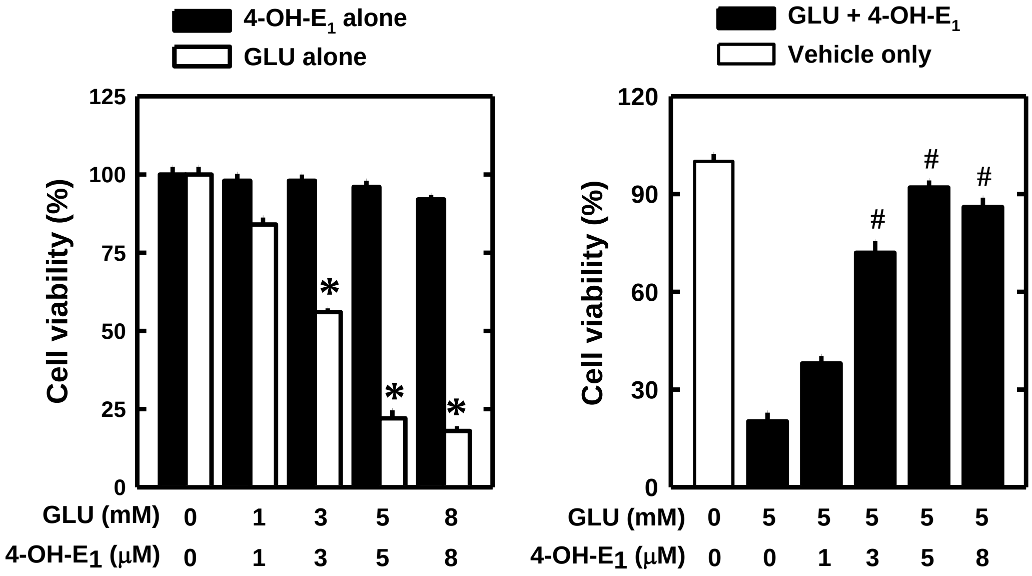

Supplementary Figure 1. Concentration dependence of 4-OH-E_1 in protecting against glutamate (GLU)-induced oxidative cytotoxicity in HT22 hippocampal neuronal cells. **(a)** Cells are treated for 24 h with different concentrations of glutamate or 4-OH-E_1 as shown. Cell viability was estimated using the MTT assay. * $P < 0.05$ versus glutamate alone. **(b)** HT22 cells are cotreated with 5 mM glutamate plus different concentrations of 4-OH-E_1 (0, 1, 3, 5, and 8 μM) for 24 h. Cell viability is estimated using the MTT assay. Each bar is mean ± S.D. of 6–8 replicate measurements. $^{\#}$ $P < 0.05$ versus vehicle only.

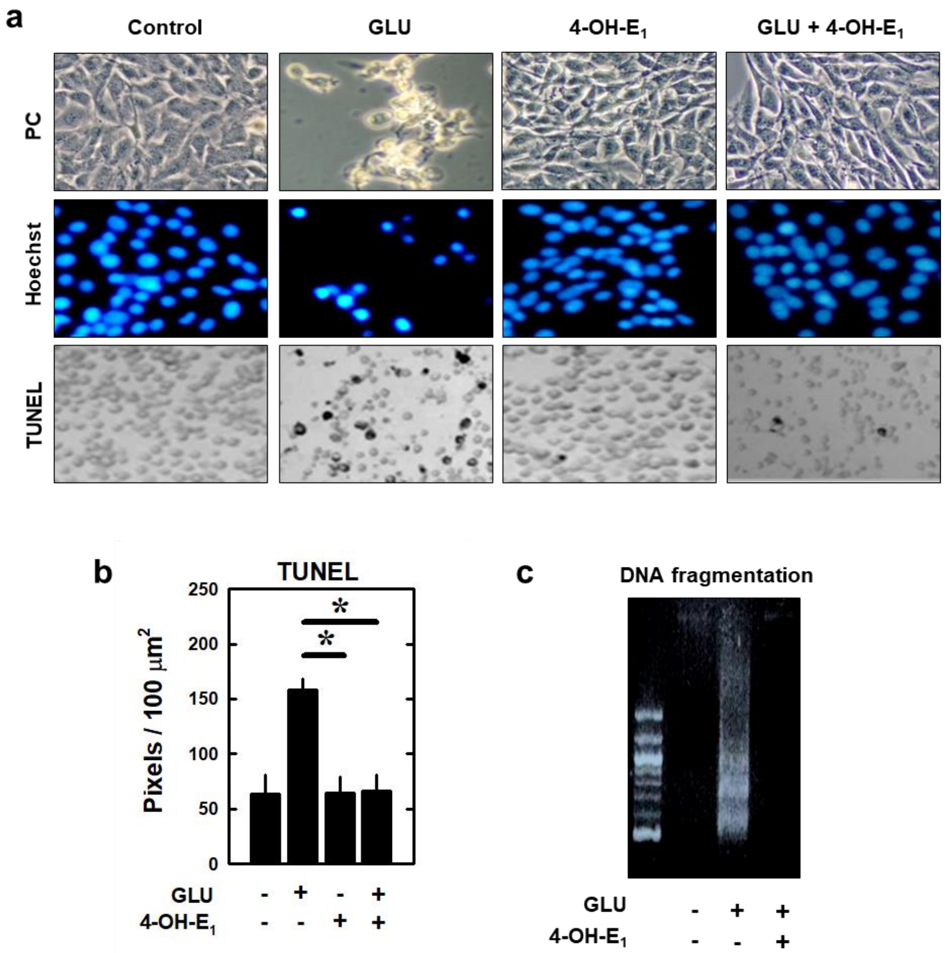

Supplementary Figure 2. 4-OH-E_1 protects against glutamate (GLU)-induced cell death in HT22 hippocampal neuronal cells (additional evidence 1). **(a)** Cells are treated with 5 mM glutamate with or without 4-OH-E_1 (5 µM) for 24 h, and then cells are stained with 10 µM Hoechst-33342 for 1 min. Cells are visualized with phase contrast microscopy (upper panels) and fluorescence microscopy for Hoechst-33342-stained cells (middle panels) and TUNEL-positive cells (lower panels). **(b)** Quantitative data for TUNEL-positive cells. Each value is the mean ± SD (n = 3). Data shown are representative of three independent experiments. * $P < 0.05$ versus glutamate alone. **(c)** DNA fragmentation patterns in different treatment groups (analyzed using the agarose gel electrophoresis).

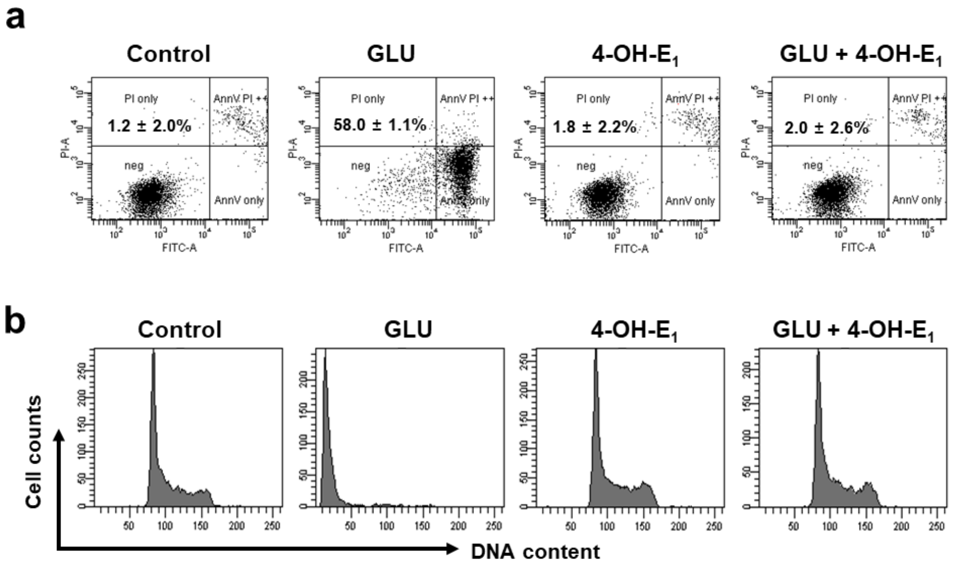

	Sub-G_1	G_0 / G_1	S	G_2 / M
Control	0.4	57.2	21.7	20.0
GLU	94.3	1.8	1.9	1.8
4-OH-E_1	0.5	52.3	21.7	25.0
GLU + 4-OH-E_1	0.5	53.5	21.9	16.2

Supplementary Figure 3. 4-OH-E_1 protects against glutamate (GLU)-induced cell death in HT22 hippocampal neuronal cells (additional evidence 2). **(a)** HT22 cells are treated with 5 mM glutamate with or without 4-OH-E_1 (5 µM) for 24 h. Cells are then stained with annexin-V-FITC and PI, and analyzed using flow cytometry. The value is the mean ± SD of three replicate measurements. **(b)** Cells are treated with 5 mM glutamate with or without 4-OH-E_1 (5 µM) for 24 h, and then stained with PI. The DNA content of the cells is analyzed using flow cytometry. A representative data set is shown (upper panel), and the relative cell populations (mean of duplicate measurements) are shown in the table below (lower panel).

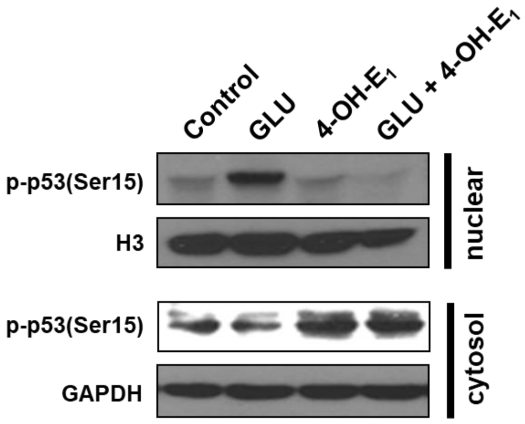

Supplementary Figure 4. Effect of 4-OH-E_1 on glutamate (GLU)-induced changes in p-p53 (Ser15) subcellular localization in mouse hippocampal neurons in primary culture. To prepare mouse hippocampal neuronal cells in primary culture, we use embryonic day 18–19 mouse hippocampus as described earlier (Belousov *et al. J. Neurosci.* **21**, 2015–2027, 2001). Neurons are plated on glass coverslips and raised in Neurobasal medium (Invitrogen) with supplements (B27 and glutamine), gentamycin (50 mg/L), and cytosine β-D-arabinofuranoside (5 μM). The culture medium is changed twice a week. The hippocampal neurons in primary culture are then treated with 5 mM glutamate alone or in the co-presence of 4-OH-E_1 (5 μM) for 24 h. Cytosolic and nuclear fractions are prepared from these cells, and the p-p53 (Ser15) protein is analyzed using Western blotting.

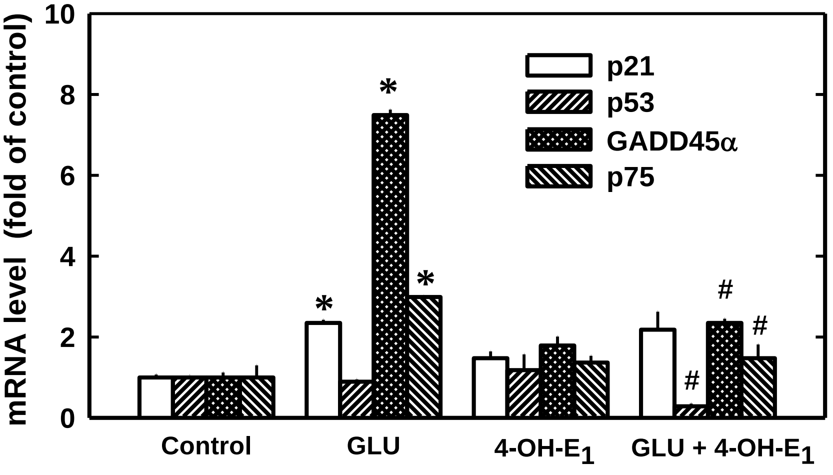

Supplementary Figure 5. 4-OH-E_1 inhibits glutamate (GLU)-induced p53 transcriptional activity in HT22 hippocampal neuronal cells. Cells are treated with glutamate (5 mM), 4-OH-E_1 (5 μM), or their combination for 24 h, and then the mRNA levels of several p53 transcriptional target genes (p21, p53, GADD45α and p75) are detected by real-time qPCR. Each bar is mean ± S.D. of triplicate measurements. * $P < 0.05$ versus the respective vehicle control groups; # $P < 0.05$ versus the respective glutamate alone groups.

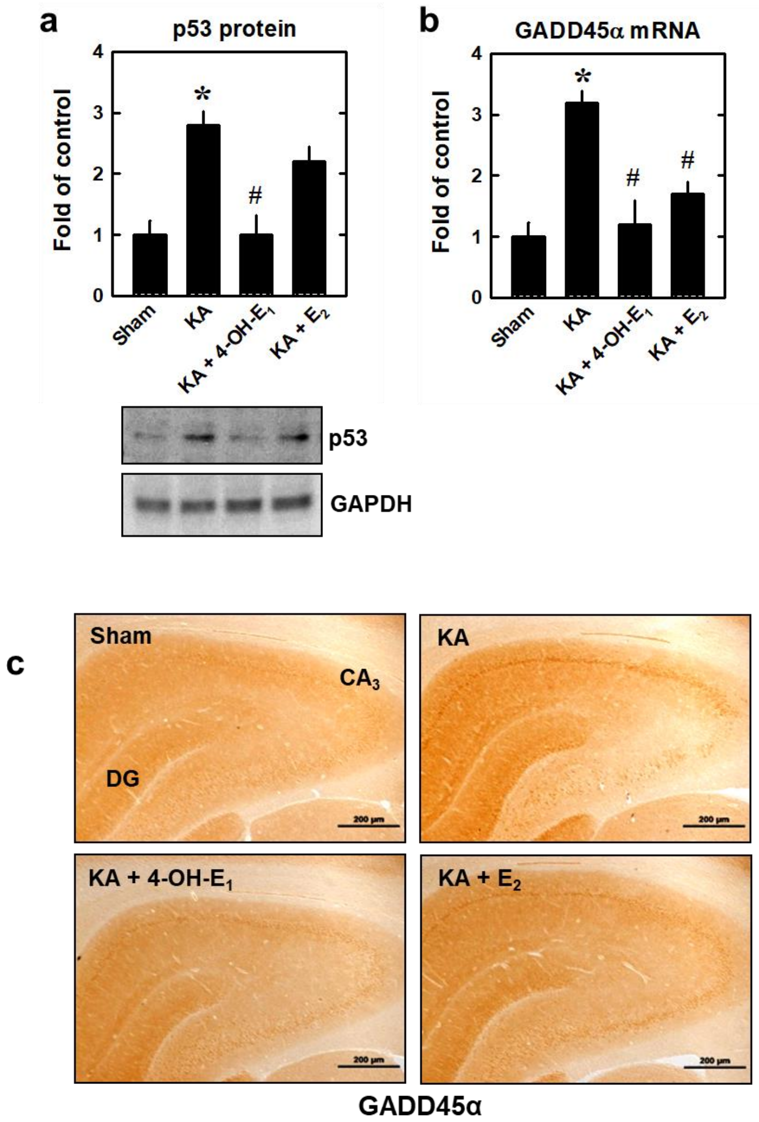

Supplementary Figure 6. Effect of 4-OH-E_1 on kainic acid (KA)-induced GADD45α expression in rat hippocampus. KA (1 µL of 1 µg/µL solution) is injected into the left and right lateral ventricles of male rats (anterior/posterior, -1.0; rostral, ± 1.6; dorsal/ventral, 4.5) using a microliter syringe under anesthesia with ketamine and xylazine (50 and 5 mg/kg, *s.c.*). The control rats (sham-operated) are injected with 1 µL of saline. **(a)** p53 protein level in the hippocampal region of rats receiving different treatments as indicated. Representative Western blots are shown in the lower panels. **(b)** The relative levels of

GADD45α mRNA in the hippocampal region of rats receiving different treatments. **(c)** Immunohistochemical staining of GADD45α protein in the hippocampal regions of rats receiving different treatments. For all the quantitative data, each value is the mean ± SD (n = 5). * $P < 0.05$ versus the sham-operated control group. $^{\#}$ $P < 0.05$ versus the KA alone group.

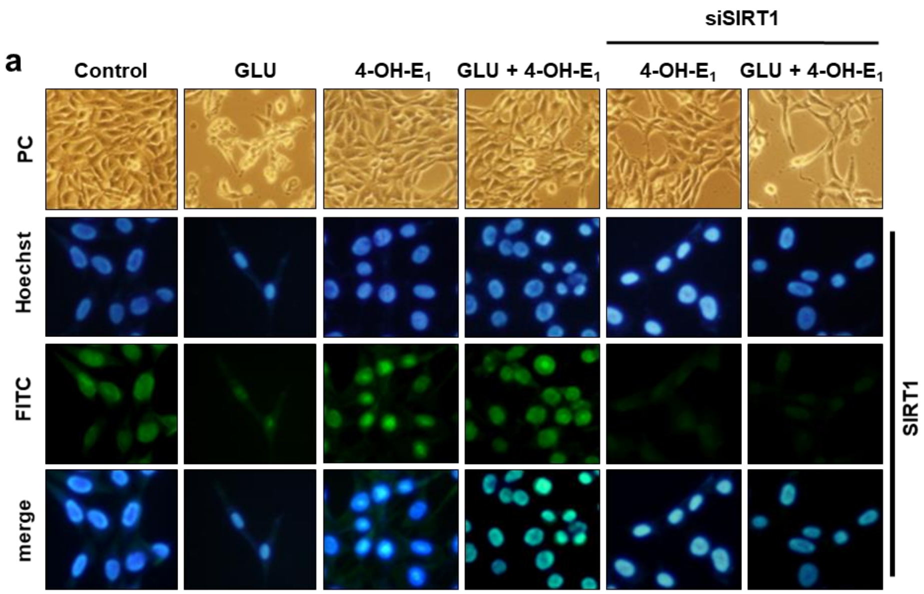

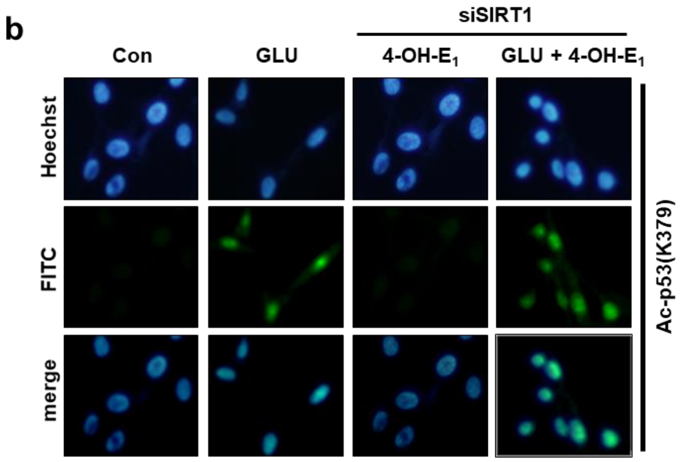

Supplementary Figure 7. Effect of 4-OH-E_1 on the cellular level and localization of SIRT1 in glutamate (GLU)-treated HT22 hippocampal neuronal cells. Cells are transfected with SIRT1 siRNA (siSIRT1), and 24 h later, cells are exposed to 5 mM glutamate alone or in combination with 4-OH-E_1 (5 μM) for additional 24 h. They are then analyzed for the immunofluorescence staining of SIRT1 **(a)** and acetylated-p53 (Lys379) **(b)**. The magnification is ×200.
